# TóMALO SUAVE (TAKE IT EASY): HOW LATINO OLDER ADULTS PERCEIVE OF POSITIVE AGING

**DOI:** 10.1093/geroni/igz038.2623

**Published:** 2019-11-08

**Authors:** Lissette M Piedra, Melissa Howe, John Ridings, Yadira Montoya, Kendon Conrad

**Affiliations:** 1 University of Illinois at Urbana Champaign, Urbana, Illinois, United States; 2 NORC at the University of Chicago, Chicago, Illinois, United States; 3 The Salvation Army Evanston Corps Community Center, Evanston, Illinois, United States; 4 the School of Public Health at the University of Illinois at Chicago, Chicago, Illinois, United States

## Abstract

With the help of the Positive Aging of Latinos Study (PALS) steering committee (N = 20), we used concept-mapping methods to learn what Latino older adults (N = 101) consider important for aging well, positively, and successfully. We used data from nine focus groups (six Spanish, three English) to generate an unabridged list of 171 statements that described what positive and successful aging meant to participants. The PALS steering committee reviewed the statements, assisted with the translation and back translation of items in Spanish, eliminated vague and duplicate statements, and approved a final list of 85 statements. Next, Latino older adults thematically sorted (n = 35) and rated (n = 93) the 85 statements (using a 1-5 scale; higher values indicate greater importance). These data were used to produce a concept map for how participants conceptualized positive and successful aging. The final map consisted of 11 clusters nested within 4 overarching regions. Region 1 [Self-Sufficiency] contains clusters of items which address “Stability” and “Independence.” Region 2 [Healthy Behaviors] includes clusters with items related to “Staying Healthy” and “Avoiding Trouble.” Region 3 [Perspectives on Life] encompasses four clusters of items that address mindsets: “Tómalo Suave (Take it Easy),” “Outlook on Life/Self-Care,” “Emotional Well-being,” and “Maturing.” Region 4 [Convivir (To coexist)] features indictors of interrelatedness such as “Social & Community Engagement,” “Coping & Adjustment,” and “Family Relationships.” These findings provide insights into how Latino older adults conceive of positive aging, which could be useful when designing culturally sensitive programming for Latino seniors.

